# Preventive Medicine via Lifestyle Medicine Implementation Practices Should Consider Individuals’ Complex Psychosocial Profile

**DOI:** 10.3390/healthcare10122560

**Published:** 2022-12-17

**Authors:** Zacharias Papadakis, Andreas Stamatis, Matthew Manierre, Ali Boolani

**Affiliations:** 1 Human Performance Laboratory, Department of Health Promotion and Clinical Practice, Barry University, Miami Shores, FL 33161, USA; 2 Exercise and Nutrition Sciences, State University of New York, Plattsburgh, NY 12901, USA; 3 Department of Humanities and Social Sciences, Clarkson University, Potsdam, NY 13699, USA

**Keywords:** fatigue, sleep, mental workload, physical activity, polyphenols, caffeine

## Abstract

Noncommunicable chronic diseases are associated with lifestyle behaviors. Psychological and social factors may influence the adoption of such behaviors. Being mentally and physically energized or fatigued may influence the intention–behavior gap of healthy lifestyle adoption accordingly. We investigated the associations of age, sex, lifestyle behaviors, mood, and mental and physical energy and fatigue at both the trait and state levels. The participants (*N* = 670) completed questionnaires assessing their sleep, mood, mental and physical state energy and fatigue, physical activity, mental workload, and diet. The ordinary least squares regression models revealed an overlap between the mental state and trait energy levels for males who consume polyphenols, have a high mental workload, and sleep well. Being younger, having a high stress level, bad sleep habits, and being confused and depressed were associated with high mental fatigue. Physical energy and fatigue shared the same commonalities with the previous results, with greater discrepancies observed between the state and trait indicators compared to that between mental energy and fatigue. Diet and stress management seem to be predictors of high physical energy, and females report higher physical fatigue levels. Health care professionals should consider this psychosocial complex profiling in their differential diagnosis and when one is implementing lifestyle behavioral changes to address the facets of preventive medicine, wellness, and health promotion.

## 1. Introduction

Lifestyle medicine (LM) and associated lifestyle behaviors, such as whole food, plant-based diet, physical activity, sleep, stress management, healthy relationships, and avoiding harmful substance use, are advocated as therapeutic approaches to prevent and treat lifestyle-related noncommunicable chronic diseases [1,2]. By addressing and ameliorating the underlying causes of the aforementioned lifestyle behaviors and negative emotional states, physical, social and emotional health well-being may be achieved [3,4,5].

Physical, social, and emotional health well-being are areas that are examined by positive psychology, where it is acknowledged that, in various settings, populations and lifestyle-related diseases, being healthy, and having optimal functioning requires more than the absence of illness [6,7,8]. Implementing positive psychology practices in healthcare may play a significant role in addressing and/or ameliorating the noncommunicable chronic diseases contributing to a healthy status [8,9]. Such a practice may be an additional health asset to the traditional health care provided by the primary care physicians in accordance with the lifestyle pillars of the lifestyle medicine education (LMEd) [10,11] model to improve the person’s emotional, social health, and entire well-being [11,12,13,14,15].

When the person’s entire well-being is examined in a health care settings, about twenty percent of the individuals seeing their primary care physician report symptoms of fatigue [16,17]. People report fatigue mostly as physical and mental manifestations, but also the inability to perform tasks/physical challenges and a delayed recovery following such activities [18]. A variety of fatigue definitions exist. In fact, there is a difference between how healthy and diseased individuals (e.g., those with anemia, thyroid disease, renal failure, cancer, malnutrition, chronic fatigue syndrome, heart failure, fibromyalgia, chronic obstructive pulmonary disease, multiple sclerosis, amyotrophic lateral sclerosis) describe fatigue [19]: healthy individuals describe it as a normal response to prolonged intense activity, while diseased individuals describe it as tiredness at rest and a lack of energy [20]. In addition, there is a sex discrepancy regarding how fatigue is described, as males describe fatigue as a feeling of being tired, while females describe it as a feeling of depression and anxiety [21,22], with increasing age being a debilitating factor [19,23,24]. Concerning sleep, patients who do not sleep well report fatigue as lack of energy and being mentally exhausted with a delayed recovery after physical exertion [25]. Irrespectively of the cause, daily physical activity, social interactions, and work stress management have been shown to improve fatigue [17,18]. Therefore, fatigue, in summary, exhibits the symptomology of a lack of energy and exhaustion, [19] and it is a multidimensional construct [26,27] (i.e., it is emotional, behavioral, and cognitive), and it has a negative health impact as it disturbs sleep and impairs social relationships, anxiety, depression, and quality of life [19]. However, recent work suggests that fatigue may in fact not be a lack of energy, but that instead, energy and fatigue are two distinct unipolar moods with their own biological correlates [28,29,30] that express themselves through human movement [31,32,33,34].

For the purposes of this study, we conceptualized energy and fatigue as two distinct unipolar moods with their own mental and physical trait and state moods [28,33,35,36,37,38,39,40,41,42,43,44]. Briefly, short-term, transient feelings of energy and fatigue are defined as state mood or state affect in psychology literature [45,46,47,48], while long-term, stable affective responses are called trait mood or trait affect [37,49]. Trait and state self-control behaviors can independently predict lifestyle healthy behaviors in relation to diet, smoking, and physical activity, especially when these concepts are examined in terms of the intention–behavior gap [50]. Physical inactivity is a state of clinical significance that disturbs the health status of an individual [51] to the extent that it can be classified as a behavioral disorder [52]. Performing many self-control trait-associated behaviors is related to less tobacco use and a lack of eating disorders [53]. Dietary habits including a plant-based diet, polyphenol, carbohydrate, and alcohol consumption have been linked to feelings of energy or fatigue [54,55,56,57] and trait and state mindful eating positively predicts a healthier eating behavior that leads to healthier habits and weight management [58]. Sleep quality and quantity have been consistently found to be correlated with decreased feelings of fatigue [59,60].

Moreover, aging is associated with decreased energy availability/a lack of energy, which, in turn, is associated with a decrease in the physical activity levels, and is it moderated by cognitive function [61]. Furthermore, due to mental fatigue, cognitive function is reduced which influences physical/balance performance [62]. Individual engagement at the self-regulation level with resistance to temptations and persistence in the face of failure and discouragement to overcome life’s adversities has been proposed as habitual responses which prevent ill-health and improve well-being [63,64]. Failure to properly self-regulate is possibly due to them having insufficient energy [64,65]. In summary, a lack of energy or fatigue has affective, motivational, and cognitive implications which negatively influence people’s quality of life [66,67]. Fatigue, as a concept, incorporates subjective feelings and objective, measurable decrements of performance [68,69,70].

Physicians do not have a well-accepted clinical criterion regarding when or how to diagnose fatigue [71]. General practitioners (GPs) address fatigue under co-occurring symptomatology, and they take a wait-and-see perspective, making the entire process really challenging. Such a practice, though it creates problems such as a poor prognosis, will devalue the severity of the case and it will affect the proper management/treatment [18,72]. Therefore, it is imperative for the health care professional to identify any prognostic factors early (e.g., age, sex, sleep, mood, stress, physical activity, and diet) in order to predict the course of the fatigue and better facilitate the subsequent medical actions. Currently, the majority of the literature has focused on the fatigue prognostic factors of selected patients [73,74,75,76], with there being a lack of research on primary care and on the general population [74,77,78]. Recently, lifestyle and behavioral therapies have been proposed as alternative approaches to treating fatigue, but the multidimensionality of fatigue and its related associations with all of the involved factors make its applicability challenging for health care practitioners [79,80,81].

For instance, if fatigue is misdiagnosed and improperly treated, it can be escalated to chronic fatigue syndrome (CFS), which can be a financial burden as it is associated with increased health care costs. Consequently, it is important to diagnose fatigue early, plan effective interventions, and evaluate the treatments’ effectiveness [82]. Including this complexity in the prognostic process of the GPs, when they are visiting with patients that report fatigue (mental and/or physical) may assist in providing better patient-oriented health care. Therefore, the aim of this exploratory study was to identify the complex associations of age, sex, lifestyle behaviors (e.g., sleep, diet, and physical activity), and mood with mental and physical energy and fatigue at both the trait and state levels. Due to the complexity of the possible associations between the examined variables, a concrete hypothesis cannot be formulated. In general, we expected to see differences among age, sex, lifestyle behaviors, mood, physical activity at both the strait and state manifestation levels in respect to perceived fatigue and/or energy.

## 2. Materials and Methods

### 2.1. Participants

This study is classified as a cross-sectional study of the survey data obtained from a larger placebo-controlled, double-blinded, within-participants, randomized cross-over project which was conducted in relation to the effects of energy shot use on mood and cognition with various outcomes and have been published elsewhere [28,36,41,83]. The primary study was registered on clinicaltrials.gov (#16-05) and approved by the Clarkson University Institutional Review Board (Approval #16-34.1). All of the participants gave their informed consent for inclusion before they participated in the study, and the study was conducted in accordance with the Declaration of Helsinki. The participants were informed that their responses may be used for a secondary analysis to explore the associations between the examined variables other than the ones listed in the clinical trial [83]. As previously described [41], from a university town with a population of about 16,000 (Potsdam, NY, USA), a snowballing approach was used to recruit the participants who were older than 18 years old (21.2 ± 5 years, Mean ± SD). The participants were asked to complete a series of screening surveys (i.e., sleep, mood, and diet) using SurveyMonkey Inc. (San Mateo, CA, USA, www.surveymonkey.com). For the purposes of this current secondary analyses, no eligibility criteria were established aside for completing the surveys, and only the procedures that were followed for the current analysis in order to answer this research question are presented. From the initial respondents (*N* = 1007) and after the data screening and removal of any outliers, for this analysis, we utilized a sample of *n* = 644–674 participants, as described in the statistical analyses and results sections. For a full list of the whole registered clinical trial and its experimental design, the interested readers may refer to Boolani et al. [41] and Boolani et al. [83], respectively.

### 2.2. Instruments

#### 2.2.1. Sleep Quality

The Pittsburgh Sleep Quality Index (PSQI) [84] was used to measure sleep quantity and quality. This 19-question survey assesses seven aspects of the respondent’s sleep in the past month, including the overall quality, the time to fall asleep, disturbances, the use of medications, and daytime dysfunction; the sleep quality, latency, and duration; habitual sleep efficiency and sleep disturbances; the use of sleep medications and daytime dysfunction over the past month. For this study, all of the component scores were summed up and reported as a global sleep quality index score, where lower scores indicate better sleep quality, while a score of five or more indicates potential sleep problems [84]. In our current study, 85% of the subjects did not answer the question “other reasons you haven’t slept” with a valid response. We removed this question from the scale and changed our calculation of the sleep disturbances component of the PSQI by revising the cut-offs from the original study, which divided the range of that scale into thirds. The final scoring for component 5 was 0 if the sum of items was 0, while it was 1 if sum of items was 1–8, 2 if sum of items was 9–16, and 3 if sum of items is 17–24.

#### 2.2.2. Profile of Mood Survey—Short Form (POMS—SF)

The 30-item POMS—SF was used to assess mood [85,86]. The respondents were asked to rate how a word/statement (e.g., How have you been feeling in the past week, including today?) described their feelings in the past 30 days on a five-point scale ranging from “Not at all” (scored as 0) to “Extremely” (scored as 4). The scores from nineteen of these questions were used to calculate the different components of tension/anxiety (α = 0.81), depression (α = 0.94), anger (α = 0.82), and confusion (α = 0.61) states. Most of the dimensions were made of 5 items, except for the confusion state, which was comprised of only 4 items. This is because a preliminary examination revealed that the inclusion of the statement “efficient” in the scoring of the confusion dimension reduced the scale alpha to 0.48. Excluding this item improved the scale reliability greatly.

#### 2.2.3. Mental and Physical State and Trait Energy and Fatigue Scales

The Mental and Physical State and Trait Energy and Fatigue scale was used to differentiate between mental and physical energy and fatigue [29,30,41,87]. The trait component, which references how the respondent usually feels, contained 12 total items, with 3 items for each of the four trait outcomes (physical and mental energy and fatigue). The representative statements included: “I feel I have energy” and “I have feelings of being worn out”. The responses were collected on a 5-point scale ranging from “never” to “always”. The state component again had the same 12 items as the trait scale, but this time, they were measured on from 0 to 100 on a Visual Analog Scale (VAS), and the time scale was used as a reference for how the respondent felt in the immediate moment. However, due to limitations in our data collection process, we used a 0 to 10 Likert scale, similar to that which was used by Boolani and colleagues [29,88]. In other studies, the Cronbach’s alpha coefficients ranged from 0.82–0.93 [36,41,87,89,90]. Within the current data, the alpha coefficients ranged from 0.73 to 0.93.

#### 2.2.4. Perceived Mental Workload

The fatigue-related background questions from the Mental and Physical State and Trait Energy and Fatigue questionnaire collected information on the perceived mental workload of the respondent during both work and off-work days [29,90,91]. The work day mental load was assessed as the number of days the participant was at school/work, the number of hours spent on their work per day, and the self-rated intensity of the mental work performed. The intensity was rated using the following scale: 1 = very low intensity; 2 = low intensity; 3 = average; 4 = intense; 5 = very high intensity. Assuming that they had at least 4 h of sleep per night, the scores ranged from 0 to 700. These three factors were multiplied together to yield an overall measure of the mental workload. The off-work day mental load was measured with the same set of three questions, which were rewritten to reference the mental work that was performed on non-work/non-school days.

#### 2.2.5. Self-Reported Physical Activity

Physical activity was also calculated using the questions from the Mental and Physical State and Trait Energy and Fatigue questionnaire [90,92]. The respondents were asked to separately report the number of hours per day and the number of days per week that they performed a high- and moderate-intensity activity. The estimated hours per week spent performing a high/moderate-intensity activity was calculated from this information, and we subtracted 168 from it (i.e., 7 days × 24 h = 168 h per week), which yielded the number of low/no activity hours per week. The number of hours per week spent performing a high-intensity activity was multiplied by 9, and the number of hours spent performing a moderate-intensity activity was multiplied by 5. The number of weekly hours that did not involve high- or moderate-intensity exercise was multiplied by 1. These weightings are based on the approximate oxygen consumption required for each exercise intensity level, and they are also metabolic equivalents (METS). The scores were then added to determine the usual physical activity composite score.

#### 2.2.6. Polyphenol Consumption

Polyphenol consumption was measured using the food frequency questionnaire [88]. The subjects were asked to provide a serving size (small, medium, or large) and the number of times per week/month (i.e., 0–1 times per month, 1–3 times per month, 1 per week, 2–4 times per week, or 5 or more times per week) they consumed 19 different fruits, 22 different vegetables, 9 beverages containing polyphenols, as well as 6 chocolates with polyphenols. For this analysis, 0–1 times per month was coded as 0 servings, 1–3 times per month was coded as 1 serving, 1 per week was coded as 4 per month, 2–4 times per week was coded as 8 servings, and 5 or more times per week was coded as 20 servings. We then multiplied this estimate of frequency by the serving size, which was coded as a value of 1 for small servings, 1.5 for medium ones, and 2 for large ones. In total, the final scale estimated the total polyphenol consumption during one month.

#### 2.2.7. Caffeine Consumption

The subjects reported how many times per week they consumed 13 types of coffee, 10 types of tea, 34 soft drinks, 16 energy drinks, 7 different frozen desserts containing caffeine, 5 chocolate products that contain caffeine, and 4 over the counter drugs with caffeine, and there was an open-ended option to list any other items they consumed that may contain caffeine [92]. They also reported the typical amount they consumed. The amount of caffeine consumed was adjusted to reflect the serving sizes. For instance, drinking 16oz of a particular energy drink would be coded as two servings of caffeine, since a single serving is actually 8oz. The final scale estimated the number of servings of caffeine consumed per week.

### 2.3. Statistical Analyses

#### 2.3.1. Preliminary Analyses

The data were exported from Surveymonkey.com as an Excel file, which was then imported into Stata 14 for all of the analyses and data preparation. The univariate statistics and distributions of each variable were carefully examined statistically and graphically. These revealed numerous skewed distributions among the independent variables. To account for this, the skewed continuous variables were Winsorized on the right-hand side [93]. This involved taking the extreme values and replacing them with a value that corresponds with a certain percentile of the original distribution. Most of the continuous variables were rescaled to recode the values past the 95th percentile to the value of the 95th percentile, however, the BMI and PSQI scores used the 99th and 98th percentiles, respectively, because their distributions were less skewed. Once this correction was made, bivariate associations between all of the variables were explored using appropriate methods such as scatter plots, Pearson’s r, cross tabulations, and comparisons of means.

The outcome variables for state/trait mental fatigue and physical fatigue also exhibited a non-ignorable amount of right-hand skew based on both of the graphs and Shapiro–Wilk’s W test for normality. This was found to bias the estimates for the preliminary regression models, which were returning non-normal residuals. To alleviate this skewness, these four variables were transformed by taking the square root. This was determined to be the most normal transformation that could possibly be performed after examining the graphs and tests of logarithmic, cubic, and cubic root transformations.

#### 2.3.2. Primary Analyses

Each of the eight outcomes was relatively normally distributed and continuous after the aforementioned transformations were performed. Therefore, the ordinary least squares (OLS) regression models were used to model the correlations, while adjusting for the potential [94]. A post hoc power analysis using G*Power [95] found that by using the most conservative model parameters that were estimated (*n* = 659; *r^2^* = 0.155), the model was still capable of detecting small effect sizes, with a power of over 99 for effect sizes of *f^2^* ≥ 0.08 at α = 0.05. Numerous methods were used to check for violations of the regression model assumptions. The residuals for all of the models were normally distributed, as it would be expected, though some evidence of heteroskedasticity was found. To account for this, Huber–White “sandwich” estimators were used to produce robust standard errors that relaxed the homoskedasticity assumption [96]. The linearity and collinearity assumptions were tested and satisfied. The outliers were identified using plots of both the Cook’s *D* and *df* beta statistics, leading to the identification of between 14 and 20 outliers in each model. The outliers for each trait/state pairing were pooled together to ensure that each pair used the same sample.

For this analysis, we were interested in seeing if there were differences in the factors that predicted the trait and state variations of each outcome. This was determined by simply examining the statistical significance, direction, and magnitude of the effects in the trait versus state models. More sophisticated methods, such as using a seemingly unrelated regression to directly test for the differences in the magnitude of the coefficients [97] are not plausible in this study. This is because the trait and state variables are based on a different set of response scales, with one ranging from 0 to 12, and the other one ranging from 3 to 30. In lieu of this, standardized coefficients are presented to depict the relative magnitude of the coefficients across the models.

## 3. Results

The participants’ characteristics are presented in Table 1. The results are presented grouped per the trait and state attributes.

### 3.1. Trait vs. State Mental Fatigue

The OLS regression models yielded many similar correlates across the models predicting mental fatigue. In particular, younger age, a high work day mental load, high PSQI scores, and both high POMSF confusion and high POMSF depression were associated with a higher level of mental fatigue (*p* < 0.05). However, POMSF anger was only associated with decreased mental fatigue in the trait indicator, but not the state measure (trait: *b* = −0.027, *t*(652) = −2.08, *p* = 0.038 vs. state: *b* = −0.017, *t*(652) = −0.77, *p* = 0.44). Conversely, POMSF tension was only associated with state mental fatigue, but not trait fatigue trait (trait: *b* = 0.011, *t*(652) = 1.12, *p* = 0.264 vs. state: *b* = 0.039, *t*(652) = 2.25, *p* = 0.03) (Table 2, Table 3, Table 4 and Table 5).

### 3.2. Trait vs. State Mental Energy

Much like the mental fatigue model, there was a great deal of overlap between trait and state energy. In this case, being a man, having a high level of polyphenol consumption, a high work day mental load, and low PSQI scores were all associated with heightened mental energy scores in both the state and trait measures. There was only one discrepancy across the two models: an increased BMI is associated with higher trait mental energy values, but not state ones (trait: *b* = 0.037, *t*(650) = 2.15, *p* = 0.03 vs. state: *b* = 0.062, *t*(650) = 1.23, *p* = 0.219) (Table 2, Table 3, Table 4 and Table 5).

### 3.3. Trait vs. State Physical Fatigue

The physical fatigue variable had a noticeably larger number of discrepancies between the state and trait indicators compared to that of mental fatigue. Commonalities did exist: having a young age, a high work day mental load, high PSQI scores, high POMS confusion scores, and high POMS depression scores were all associated with heightened physical fatigue at the 0.05 level or lower, however, several predictors were uniquely associated with trait physical fatigue. High levels of polyphenol consumption (trait: *b* = −0.000, *t*(661) = −2.07, *p* = 0.04 vs. state: *b* = −0.000, *t*(661) = −0.75, *p* = 0.46) and caffeine consumption (trait: *b* = 0.004, *t*(661) = 2.52, *p* = 0.012 vs. state: *b* = 0.003, *t*(661) = 1.06, *p* = 0.29) were both correlated with higher trait fatigue, but not with state physical fatigue. Feelings of anger were also associated with only a decreased level of physical fatigue in the trait indicator (trait: *b* = −0.023, *t*(661) = −2.13, *p* = 0.034 vs. state: *b* = 0.006, *t*(661) = 0.27, *p* = 0.79). The only significant effect that was unique to state physical fatigue was that being female was associated with a higher level of fatigue, although the trait indicator was on the verge of significance (trait: *b* = 0.057, *t*(661) = 1.70, *p* = 0.09 vs. state: *b* = 0.165, *t*(661) = 2.57, *p* = 0.01) (Table 2, Table 3, Table 4 and Table 5).

### 3.4. Trait vs. State Physical Energy

Being male, highly active, and having good sleep quality were all associated with heightened physical energy levels in both the state and trait models. At the same time, physical energy had the largest number of discrepancies across the trait and state models, with five covariates being significant in only trait or state. Most of these results were unique to a high level of state physical energy, which was associated with being older than 23 years old (trait: *b* = 0.057, *t*(645) = 0.28, *p* = 0.78 vs. state: *b* = −1.89, *t*(645) = −3.48, *p* = 0.001), a higher level of polyphenol consumption (trait: *b* = 0.002, *t*(645) = 1.54, *p* = 0.124 vs. state: *b* = 0.008, *t*(645) = 2.35, *p* = 0.02), a lower level of caffeine consumption (trait: *b* = −0.008, *t*(645) = −0.95, *p* = 0.343 vs. state: *b* = −0.043, *t*(645) = −2.06, *p* = 0.04), and low work day mental loads (trait: *b* = 0.002, *t*(645) = 1.76, *p* = 0.078 vs. state: *b* = 0.010, *t*(645) = 2.94, *p* = 0.003). In contrast to this, the physical energy trait had only one unique covariate: the off-work day mental load (trait: *b* = 0.014, *t*(645) = 2.49, *p* = 0.013 vs. state: *b* = −0.007, *t*(645) = −0.46, *p* = 0.643) (Table 2, Table 3, Table 4 and Table 5).

## 4. Discussion

Drawing from the interdisciplinary area of medicine and health care research, we provide evidence on the isolated and combined effects of the psychological and social factors on lifestyle behaviors and noncommunicable chronic diseases. In more detail, the purpose of this study was to examine if there were unique predictors of trait and state mental and physical energy and fatigue associated with lifestyle behaviors which can add a prognostic value to the GP’s practices of lifestyle medicine. Based on the complex associations between the lifestyle behaviors, social, age, and sex characteristics, it was expected that we would detect differences across the predictors due to the physiological differences in the ways in which trait and state manifest themselves. Moreover, it was also expected that distinct social roles might result in differential risk exposures and lifestyle adaptations to perceived fatigue or energy problems. While all of the four pairs of trait/state predictors yielded similar results, each model revealed correlates that were specific to either the state or trait variations. This implies that though the prior research has neglected the distinction between the trait and state correlates, it may be clinically and theoretically important for the GPs to further explore both of these forms of energy and fatigue in terms of early diagnoses, effective prevention, and following up/evaluating the treatments’ effectiveness [72,82].

In summary, we reported that being younger, having high mental work day loads, being a bad sleeper, and having high confusion and depression POMSF scores were associated with high levels of trait and state mental fatigue. These findings are in line with those of the previously published literature which support the association between fatigue and mental workload [98], poor sleep [99], and depression [100]. Males who consume a diet that is rich in polyphenols, have high mental work day loads, and sleep well reported having a high mental energy level along both the state and trait mood scales. In addition to that, higher BMI values were associated with only higher trait of mental energy levels. Physical fatigue shared the same characteristics with mental fatigue, however, a diet that is rich in polyphenols and caffeine was associated with higher levels of trait fatigue, but not with state physical fatigue. This finding suggests that individuals who are normally fatigued maybe try to compensate for the chronic feelings of physical fatigue by consuming caffeine and/or other polyphenolic foods which are known to have anti-fatiguing effects [101,102]. State physical fatigue was associated with being female, while trait physical fatigue is marginally non-statistically significant in relation to this. On the contrary, being a physically active, a good sleeper, and male was associated with high levels of both state and trait physical energy. Physical energy presented different covariates regarding the state or trait: a high level of state physical energy was associated with age (>23 years old), high polyphenol and low caffeine consumption levels, and low work day mental loads, while a high level of trait of physical energy was associated with off-work day mental loads. These results may suggest that individuals who normally feel physically energetic (a high level of trait physical energy) may be more likely to perform mental work on off-work days because they feel more “up to” performing mental work on their days off.

As previously stated, age is a correlate of both trait and state mental and physical fatigue, which is in line with the majority of the literature that suggests that younger individuals (<23 years old) feel more fatigued [103,104,105]. We also demonstrated this across both trait and state fatigue, whilst also distinguishing between mental and physical energy, as this difference is important because neither mental energy nor trait physical energy were correlated with age. It is possible that the concepts of state and trait and their interactions in different situations or contexts which exist due to hereditary influences and environmental factors may have affected the reported frequency and intensity of the mood states [37].

The current study does not have the indicators that are needed to identify the specific causal mechanisms that drive these differences, however, the explanations may lie in either differences in the physical characteristics of the brain (e.g., central governor, cerebellar development, neurogenesis, elevated trophic factors, and the vascularization of the hippocampus) [106,107] or the combination of role transitions, turbulent schedules, and high-pressure environments faced by the college students who comprised most of the younger age bracket for this study [108]. Brain differences have been also suggested by numerous studies examining chronic fatigue syndrome, as the patients tended to have high trait fatigue and low trait energy levels [109]. Several functional magnetic resonance imaging studies have found differences in brain functioning among the people with chronic fatigue syndrome versus those without it [110,111,112]. For instance, De Lange et al. observed inactivity in the ventral anterior cingulate cortex in patients with chronic fatigue syndrome when they made an error while they were performing mental tasks [110]. Therefore, the physiological differences between the individuals who exhibit high trait fatigue levels might better be construed as a manifestation of persistent dysfunction, versus state fatigue and energy, which might reflect a shorter reaction time to proximate stimuli.

Moreover, fatigue and energy appear to be two separate, albeit correlated, constructs [113]. Even though energy and fatigue are assumed to be the two ends of the same continuum, evidence exists which suggests that certain interventions impact either energy or fatigue only, suggesting that these mood states may be distinct as well [59,114,115,116,117,118,119]. Therefore, since in this study we operationally defined energy and fatigue as two separate mood traits and states, this may have influenced our results. We decided to proceed with this nuance, as this would add value to the conceptual precision of the study, allowing us to identify the possible interventions and determinants that are specific only to fatigue and/or energy at the same time. For example, Nozaki et al. reported distinct biochemical differences between mental and physical fatigue states [120]. Likewise, the work by our group found unique predictors for both mental and physical energy and fatigue traits [28,29,31,38,39,41,42,83,88,101,102]. Our results provide insights to the complexity that surrounds trait/state and energy/fatigue in relation to the mental/physical elements, perceived mental work on non-work days, total time spent sitting, and global sleep quality [41]. Under this perspective, sleep quality and quantity are a particularly consistent, and they are typically correlated with decreased feelings of fatigue [59,60], while high levels of physical activity are correlated with increases in both energy and fatigue, as evidenced by a meta-analysis with 6807 subjects spread over 70 studies [121]. Since the majority of our sample consisted of college students, this is also of high importance for the health care practitioners, as mental health issues among college students have been consistently increasing, with feelings of fatigue and low energy levels being reported [122]. Female college students report more feelings of mental or emotional fatigued compared to those of males, which is something that impacts not only their quality of life with increased stress and anxiety, but also their academic performance [123]. When the sleep quality is added as a factor, females report poor sleep quality [124], which may contribute to the feelings of fatigue and low energy in this population.

We present that sex differences influence trait/state energy and fatigue, as previously reported, as female respondents tend to report higher levels of fatigue states than males do [125]. It was ascertained that such a phenomenon is based on the biological or social differences between the sexes, but this was not this study’s purpose. However, it is possible that the socio-demographic factors of sex/age-specific roles influenced the reported trait/state energy and fatigue levels [36,126]. For example, older individuals experience less state fatigue as the reported changes to the brain restructuring of females demonstrates that they have greater fatigue resilience compared to males [105]. Additionally, single females between 30–44 years old and of lower educational attainment reported high fatigue levels [104]. These sex differences may be due to our sampling strategy and received responses (e.g., only 38% of them were females), however, it may reflect on the sex-specific differences regarding how fatigue or illness is experienced in both the clinical and non-clinical settings [19,24,127]. Hormonal, stress-related factors, the social context, and the roles that they play have been proposed as possible explanations for this sex difference [27,78,79,105,128].

It is in line with the literature that being physically active is associated with feelings of high physical energy and low physical fatigue levels [129]. Previous work from our group has reported that aquatic exercise improved feelings of physical and mental energy, with social functioning being a determinant of mental and physical fatigue [35]. Even though in this study we did not directly examine sitting time, it has been reported that physical activity is associated with feelings of mental and physical energy and mental fatigue for individuals with a sedentary lifestyle (i.e., sitting time < 8 h) [39].

In contrast with our results of physical activity and sleep quality, Herring et al. found no differences when they compared feelings of energy of good sleepers vs. poor sleepers using the PSQI instrument [129]. Moreover, recently, we reported that in a college student population, sleep quality was a factor for mood disturbances, indicating the importance of proper sleep hygiene practices [91], with the amount of physical activity predicting of the affect of poor sleep habits on state feelings of mental/physical fatigue/energy [130]. Such a discrepancy may be attributed to the fact that we removed a question from the survey due to the fact that only 25% of them answered it, altering, therefore, the respective calculations of the PSQI components by revising the cut-offs, as originally proposed [84].

In addition, our results regarding dietary habits, and especially polyphenol and caffeine consumption, provide correlates for feelings of energy and/or fatigue [55,56,57]. A diet that is rich in polyphenols (e.g., cocoa flavanols) improved the feelings of mental fatigue, and even though a clear mechanistic explanation is not known, it is postulated that it is related to the known vasodilatory effects and improvements in endothelial function due to the polyphenols [131,132,133,134,135,136,137]. Caffeine has been used as a remedy for low energy and high fatigue levels [138,139], with the inter-individual differences reporting on the effect of caffeine on moods [140,141,142,143]. ADORA2A gene polymorphism has been linked to the effects of caffeine use and inter-individual differences on anxiety and physical fatigue [144,145,146].

This study provides valuable insight to physicians as it assesses sleep quality, physical activity, diet, workload, mood, and feelings of fatigue and energy, simultaneously. Such an attempt allows the physicians to account for each of these health-related behaviors when they are examining their associations under the pillars of LM. Our robust sample size and the statistical analyses allowed us to estimate all of the associations simultaneously while also minimizing any possible errors in the analyses.

Measuring effects, moods, and emotions in health behavioral research is not free of limitations, as there are fundamental constructs that affect their respective measurements [147]. This study used POMS—SF [85], not only because it is the most popular self-reported measure in exercise, sport, and psychology, and health behavioral research [148,149,150,151], but also because we wanted to capture Fatigue–Inertia states as a factor from the structures of Tension–Anxiety, Depression–Dejection, Anger–Hostility, Vigor–Activity, and Confusion–Bewilderment states [152]. We attempted to represent the LM pillars more accurately and holistically by focusing on mental and physical fatigue, while accounting for sleep, stress, diet, and physical activity [10,14,15,41,153,154,155]. It has to be noted that even though POMS defines six mood factors, this structure becomes apparent because of the selection of the specific adjectives that were used, with there being a possibility for a different mood profile to emerge if other adjectives had been included by the McNair et al. [147,152]. Another issue that relates to POMS—SF has to do with the bipolar dimensionality of the form which is prone to measurement errors because its unipolar response format is subject to an extreme response bias, resulting in some bipolar and unipolar emerging factors, with a circular order of mood states being present [156]. Related to use of the POMS—SF is the fact that we used it to calculate physical activity from the Mental and Physical State and Trait Energy and Fatigue questionnaire. We recognize that such a practice failed to capture the unique exercise-related stimuli, since POMS is heavily skewed towards measuring negative scales, and due to its applicability beyond the college age [157]. Since the notion that heath is not just the absence of negative feelings but also the presence of positive feelings of well-being, the use of the Subjective Exercise Experiences Scale (SEES) may have been more appropriate to capture the balanced responses in relation to exercise stimuli [147,158,159], but POMS—SF was chosen for the participants’ convenience and its relation to the main variables of interest. Therefore, our POMS—SF results cannot be generalized to the global domain of mood, but it can only be generalized to the distinct ones assessed by POMS—SF, since there is not an encompassing sense of the global mood domain [147].

In addition, physical activity was calculated using questions from the Mental and Physical State and Trait Energy and Fatigue questionnaire, as reported in the methods section, however, self-reported measures are known to be subject to biases that may influence the observed relationships [160]. Despite this limitation, the results reflect prior research that links physical activity with stress, sleep quality, and physical fatigue [161,162,163,164,165].

Moreover, since this is a cross-sectional study, casual relationships cannot be inferred. Therefore, future research should utilize longitudinal data to examine the aforementioned relationships. Related to our population, females comprised 38% of our sample, which is something that is dissimilar from the 72% female population who completed nationwide surveys regarding college student health [122]. However, this study was primarily conducted at an engineering school in upstate New York, and the study population reflects the male: female ratio at this particular university. Future studies need to ensure that there is equal participation between the sexes for adequate representation and results extrapolation. Moreover, for the patient of the future, the use of wearable technology to collect accurate sleep, diet, physical activity data may be the key to LM adoption in the health care field, as more relevant data can be evaluated by the GPs during regular checkups, and individualized preventive medicine can be prescribed [166,167]. Nevertheless, our results can be utilized with caution by healthcare professionals in their efforts to implement behavioral changes to their patients. Fatigue treatment has been both challenging and often overlooked by GPs due to a lack of specificity regarding diagnostic procedures [168]. This study adds value to the numerous fatigue instruments that have been used for specific diseases [168] by differentiating the fatigue as a feeling that is experienced by the community and the applicability of the questionnaires across an apparently healthy population [169,170,171].

## Figures and Tables

**Table 1:** Descriptive statistics for continuous variables *(N* = 666).

	Mean	SD	Min	Max
**Outcomes**				
Sqrt(Mental Fatigue Trait)	2.02	0.6	0	3.46
Sqrt(Mental Fatigue State)	3.37	0.94	1.73	5.74
Mental Energy Trait	5.82	2.02	0	12
Mental Energy State	17.35	5.98	3	33
Sqrt(Physical Fatigue Trait)	2.19	0.46	1	3.61
Sqrt(Physical Fatigue State)	3.31	0.89	1.73	5.48
Physical Energy Trait	6.5	2.16	0	12
Physical Energy State	18.01	5.71	4	33
**Covariates**				
% 22 or younger	83			
% Female	38			
BMI	24.15	4.39	14.8	39.62
Physical activity score	225.85	36.24	168	310
8oz Caffeine servings/week	12.54	9.78	1	41
Polyphenol consumption	95.45	61.3	5	299
Work days mental load	115.63	59.91	9	240
Off-work days mental load	18.76	14.52	0	56
Sleep Quality Score	5.03	2.54	0	17
POMS Anger	6.69	1.97	5	12
POMS Confusion	5.73	1.74	4	10
POMS Tension	7.8	2.69	5	14
POMS Depression	6.86	2.31	5	13

**Table 2:** Comparing predictors of trait and state mental energy and fatigue.

	Mental Fatigue (*N* = 666)				Mental Energy (*N* = 664)			
	Sqrt(Trait)		Sqrt(State)		Trait		State	
Variables	Coef	*β*	Coef	*β*	Coef	*β*	Coef	*β*
22 or younger (ref: 23 or older)	0.101 *	0.069	0.247 **	0.101	0.019	0.004	−0.908	−0.057
Female (ref: male)	0.049	0.042	0.107	0.056	−0.456 **	−0.110	−1.626 ***	−0.132
Physical Activity Score	0.001	0.063	0.000	0.006	0.000	0.005	0.007	0.043
BMI	−0.001	−0.012	0.008	0.040	0.037 *	0.080	0.063	0.046
Polyphenol Consumption	0.000	0.020	−0.000	−0.032	0.006 ***	0.190	0.013 ***	0.133
Caffeine Consumption	0.002	0.042	0.003	0.035	−0.006	−0.029	0.000	0.000
Work Day Mental Load	0.001 *	0.085	0.002 **	0.110	0.003 *	0.080	0.009 *	0.092
Off-work Day Mental Load	0.001	0.026	0.002	0.028	0.009	0.061	−0.003	−0.008
Sleep Quality Score	0.075 ***	0.327	0.093 ***	0.245	−0.210 ***	−0.250	−0.704 ***	−0.284
POMS Anger	−0.027 *	−0.096	−0.017	−0.037	0.093	0.090	0.263	0.087
POMS Confusion	0.067 ***	0.207	0.089 ***	0.166	−0.112	−0.096	−0.063	−0.018
POMS Tension	0.012	0.055	0.040 *	0.114	−0.027	−0.036	−0.154	−0.069
POMS Depression	0.040 **	0.165	0.052 *	0.130	−0.060	−0.069	−0.170	−0.066
Constant	0.642 ***		1.111 ***		5.719 ***		17.870 ***	
R-squared	0.304		0.265		0.155		0.148	

* *p* < 0.05, ** *p* < 0.01, *** *p* < 0.001.

**Table 3:** Comparing predictors of trait and state physical energy and fatigue.

	Physical Fatigue (*N*= 674)				Physical Energy (*N*= 659)			
	Sqrt(Trait)		Sqrt(State)		Trait		State	
Variables	Coef	*β*	Coef	*β*	Coef	*β*	Coef	*β*
22 or younger (ref: 23 or older)	0.095 *	0.080	0.233 **	0.100	0.058	0.010	−1.889 ***	−0.128
Female (ref: male)	0.059	0.063	0.170 **	0.093	−0.344 *	−0.078	−1.325 **	−0.115
Physical Activity Score	0.000	0.011	0.000	0.017	0.018 ***	0.304	0.035 ***	0.225
BMI	0.003	0.028	0.011	0.053	−0.021	−0.043	0.010	0.008
Polyphenol Consumption	−0.001 *	−0.072	−0.000	−0.024	0.002	0.058	0.008 *	0.088
Caffeine Consumption	0.004 *	0.090	0.003	0.036	−0.008	−0.036	−0.043 *	−0.076
Work Day Mental Load	0.001 *	0.075	0.001 *	0.091	0.002	0.065	0.010 **	0.108
Off-work Day Mental Load	−0.000	−0.004	−0.001	−0.019	0.014 *	0.091	−0.007	−0.017
Sleep Quality Score	0.042 ***	0.224	0.087 ***	0.240	−0.176 ***	−0.198	−0.627 ***	−0.270
POMS Anger	−0.023 *	−0.100	0.007	0.015	0.028	0.026	0.072	0.025
POMS Confusion	0.060 ***	0.228	0.104 ***	0.203	−0.025	−0.020	−0.126	−0.039
POMS Tension	−0.001	−0.004	0.011	0.032	0.019	0.024	0.009	0.004
POMS Depression	0.035 ***	0.176	0.046 *	0.119	−0.071	−0.077	−0.184	−0.076
Constant	1.285 ***		1.062 ***		3.629 ***		15.378 ***	
R-squared	0.222		0.248		0.197		0.206	

* *p* < 0.05, ** *p* < 0.01, *** *p* < 0.001.

**Table 4:** Comparing predictors of trait and state mental energy and fatigue with PSQI being variables decomposed.

	Mental Fatigue (*N*= 651)				Mental Energy (*N*= 649)			
	Sqrt(Trait)		Sqrt(State)		Trait		State	
Variables	Coef	*β*	Coef	*β*	Coef	*β*	Coef	*β*
22 or younger (ref: 23 or older)	0.073	0.049	0.201 *	0.082	0.124	0.023	−0.645	−0.040
Female (ref: male)	0.079 *	0.068	0.127	0.066	−0.545 ***	−0.131	−1.887 ***	−0.153
Physical Activity Score	0.001	0.067	−0.000	−0.000	0.001	0.012	0.010	0.058
BMI	−0.001	−0.012	0.010	0.049	0.037 *	0.081	0.054	0.040
Polyphenol Consumption	0.000	0.017	−0.000	−0.022	0.006 ***	0.191	0.010 **	0.107
Caffeine Consumption	0.002	0.029	0.003	0.035	−0.002	−0.012	0.005	0.008
Work Day Mental Load	0.001 **	0.091	0.002 ***	0.117	0.003 *	0.093	0.011 **	0.109
Off-work Day Mental Load	0.001	0.019	0.001	0.021	0.007	0.053	−0.004	−0.009
**PSQI categorical**								
Subjective Quality	0.070	0.071	0.270 ***	0.165	−0.279	−0.080	−1.944 ***	−0.188
Sleep Latency	0.074 **	0.115	0.041	0.038	−0.282 **	−0.119	−0.782 **	−0.111
Sleep Duration	0.059	0.070	0.012	0.009	−0.248 *	−0.081	−0.410	−0.045
Sleep Efficiency	−0.045	−0.046	−0.133 *	−0.081	0.085	0.023	0.297	0.027
Sleep Disturbance	0.019	0.015	0.146	0.069	0.265	0.057	0.480	0.035
Use of Sleep Medication	0.039	0.043	0.060	0.039	0.055	0.017	0.189	0.019
Daytime Dysfunctions	0.192 ***	0.270	0.238 ***	0.202	−0.458 ***	−0.182	−1.203 ***	−0.162
POMS Anger	−0.018	−0.063	−0.002	−0.005	0.075	0.073	0.204	0.067
POMS Confusion	0.055 ***	0.170	0.073 **	0.135	−0.091	−0.078	−0.023	−0.007
POMS Tension	0.011	0.051	0.028	0.081	−0.031	−0.042	−0.162	−0.073
POMS Depression	0.033 **	0.133	0.044 *	0.107	−0.041	−0.047	−0.084	−0.032
Constant	0.715 ***		1.070 **		5.185 ***		16.923 ***	
R-squared	0.336		0.315		0.181		0.185	

* *p* < 0.05, ** *p* < 0.01, *** *p* < 0.001.

**Table 5:** Comparing predictors of trait and state physical energy and fatigue with PSQI variables being decomposed.

	Physical Fatigue (*N*= 659)				Physical Energy (*N*= 644)			
	Sqrt(Trait)		Sqrt(State)		Trait		State	
Variables	Coef	*β*	Coef	*β*	Coef	*β*	Coef	*β*
22 or younger (ref: 23 or older)	0.086 *	0.073	0.208 *	0.090	0.075	0.013	−1.742 **	−0.118
Female (ref: male)	0.064	0.068	0.179 **	0.098	−0.404 *	−0.091	−1.509 ***	−0.131
Physical Activity Score	0.000	0.010	0.000	0.015	0.019 ***	0.318	0.036 ***	0.235
BMI	0.003	0.029	0.011	0.052	−0.022	−0.045	0.011	0.008
Polyphenol Consumption	−0.001	−0.070	−0.000	−0.012	0.002	0.044	0.006	0.068
Caffeine Consumption	0.004 *	0.081	0.003	0.029	−0.006	−0.029	−0.039	−0.067
Work Day Mental Load	0.001 *	0.080	0.001 **	0.100	0.003*	0.080	0.011 **	0.119
Off-work Day Mental Load	−0.000	−0.006	−0.002	−0.026	0.014*	0.092	−0.005	−0.014
**PSQI categorical**								
Subjective Quality	0.083 *	0.106	0.213 ***	0.139	−0.550 ***	−0.145	−1.589 ***	−0.161
Sleep Latency	0.064 **	0.124	0.100 *	0.099	−0.350 ***	−0.141	−0.979 ***	−0.152
Sleep Duration	0.025	0.037	0.010	0.008	−0.155	−0.047	−0.509	−0.059
Sleep Efficiency	−0.014	−0.017	−0.076	−0.049	−0.109	−0.028	0.422	0.042
Sleep Disturbance	0.007	0.007	0.138	0.069	0.298	0.062	−0.017	−0.001
Use of Sleep Medication	0.009	0.012	0.019	0.013	0.260 *	0.073	−0.019	−0.002
Daytime Dysfunctions	0.053*	0.093	0.143 **	0.129	−0.074	−0.028	−0.648 *	−0.092
POMS Anger	−0.020	−0.089	0.014	0.031	0.027	0.025	0.024	0.008
POMS Confusion	0.058 ***	0.221	0.094 ***	0.184	−0.031	−0.025	−0.118	−0.037
POMS Tension	−0.001	−0.008	0.007	0.020	0.020	0.026	0.027	0.013
POMS Depression	0.031 **	0.158	0.036	0.093	−0.079	−0.084	−0.127	−0.052
Constant	1.297 ***		1.037 **		3.351 ***		14.910 ***	1.297 ***
R-squared	0.236		0.273		0.235		0.234	

* *p* < 0.05, ** *p* < 0.01, *** *p* < 0.001.

## Data Availability

Not applicable.
